# Taking Screenshots of the Invisible: A Study on Bacterial Contamination of Mobile Phones from University Students of Healthcare Professions in Rome, Italy

**DOI:** 10.3390/microorganisms8071075

**Published:** 2020-07-19

**Authors:** Domenico Cicciarella Modica, Massimo Maurici, Gian Loreto D’Alò, Cinzia Mozzetti, Alessandra Messina, Alessandra Distefano, Francesca Pica, Patrizia De Filippis

**Affiliations:** 1 School of Hygiene and Preventive Medicine, University of Rome Tor Vergata, 00133 Rome, Italy; domenico.cicciarellamodica@students.uniroma2.eu; 2 Department of Biomedicine and Prevention, University of Rome Tor Vergata, 00133 Rome, Italy; cinzia.mozzetti@uniroma2.it (C.M.); alessandra.messina@uniroma2.it (A.M.); distefano.1376645@studenti.uniroma1.it (A.D.); patrizia.de.filippis@uniroma2.it (P.D.F.); 3 Department of Epidemiology, Lazio Regional Health Service, 00154 Rome, Italy; 4 Department of Experimental Medicine, University of Rome Tor Vergata, 00133 Rome, Italy; pica@uniroma2.it

**Keywords:** cell phone, students, health occupations, fomites, cross infection, Staphylococcaceae, hygiene, epidemic

## Abstract

Mobile phones (MPs) are commonly used both in the personal and professional life. We assessed microbiological contamination of MPs from 108 students in healthcare professions (HPs), in relation to their demographic characteristics and MPs handling habits, collected by means of a questionnaire. Cultural and biochemical tests were performed, and statistical analyses were carried out. Staphylococci were present in 85% of MPs, Enterococci in 37%, Coliforms in 6.5%; *E. coli* was never detected. *Staphylococcus epidermidis* was the most frequently isolated staphylococcal species (72% of MPs), followed by *S. capitis* (14%), *S. saprophyticus*, *S. warneri*, *S. xylosus* (6%), and by *S. aureus* (4%). Heterotrophic Plate Counts (HPC) at 37 °C, ranged from 0 to 1.2 × 10^4^ CFU/dm^2^ (mean = 362 CFU/dm^2^). In univariate analysis, the male gender only was significantly associated with higher HPCs and enterococcal contamination. Multiple linear regression models explained only 17% and 16% of the HPC 37 °C and staphylococcal load variability, respectively. Developing specific guidelines for a hygienic use of MPs in clinical settings, for preventing cross-infection risks, is advisable, as well as introducing specific training programs to HP students. MPs decontamination procedures could also be implemented in the community.

## 1. Introduction

Mobile phones (MPs), and smartphones in particular, are one of the most common items people carry around with them, in both their professional and personal lives [1,2].

According to United Nations Public Administration Network (UNPAN), approximately 30% of the world’s population had a smartphone at the end of 2014 [3]. The number of cellular phones in the world has been steadily increasing in the last years—it reached almost the same number as the world population in 2017 and is expected to reach a remarkable figure of 1.5 devices per capita by the end of the current year [4].

The use of MPs occurs in hospital, by patients, visitors, and healthcare workers (HCWs) [1]. Smartphones provide better communication, and sharing of information among healthcare workers (HCWs) and between HCWs and patients; they can lead to improved quality of healthcare, especially in terms of faster communication and promoting a prompt clinical diagnosis [5,6,7,8]. Due to their many benefits, any associated risk with their use is minimized or not considered [7].

The role of fomites in the transmission of infectious diseases in healthcare institutions was extensively investigated [9]. Inanimate objects can host and carry microorganisms from the surrounding environment. These microorganisms can then be transferred to another substratum, including the human body [10]. In this regard, the infectious potential of telephones was first suggested by Aronson et al. [11]. According to the classification of Earle H. Spaulding, MPs fall into the category of non-critical items [12]. However, since the hands of HCWs play an important role in the genesis of healthcare-associated infections (HAIs), MPs can act as carriers of the hands’ microbiota, thus, representing potential reservoirs for pathogenic microorganisms [13,14,15]. Furthermore, heat generated by MPs can enhance bacterial growth [7].

MPs can be more problematic compared to other fomites—born to be transported, they can facilitate intra- and inter-wards transmission of microbial pathogens. Handling MPs during patient care procedures, HCWs could easily transmit microorganisms from patients to their mobile phones and vice versa [5]. This could facilitate the spread of potential pathogens to the community [1,7,16].

HAIs have increased significantly during the last decades, reaching a pooled prevalence of 7.6% in high-income countries and 10.1% in low- and middle-income countries. [3,12,17]. In addition, infections caused by multidrug-resistant staphylococcal and enterococcal strains are a growing problem in many healthcare institutions [16].

The WHO, the Centers for Disease Control and Prevention (CDC) and the Association for Professionals in Infection Control and Epidemiology (APIC), recommend hand hygiene as the main standard procedure for the control of transmission of infectious agents and the prevention of HAIs [3]. It was estimated that up to 1/3 of all HAIs could be prevented by hand hygiene [18].

Furthermore, MPs were proposed to act as “Trojan horses” in propagating pathogens, including viruses, during epidemics and pandemics [19] Our study was aimed at evaluating the microbial contamination of MPs from students of various healthcare profession degree courses at the Tor Vergata University (Rome, Italy), all of whom were actively frequenting medical or surgical units as a part of their professional training.

## 2. Materials and Methods

### 2.1. Study Sample

The study included a convenience sample of 108 students from Tor Vergata University, Degree Courses in Healthcare Professions (Nursing, Midwifery, and a Healthcare Management Master Class), attending their internship at Health Care Facilities affiliated with the same University.

Enrollment was on voluntary basis and was strictly anonymous. After having received an exhaustive information about the research purposes, students willing to participate in the study were asked to fill in a short anonymous questionnaire and to provide their own MPs for microbiological analyses. Informed consent was obtained from each participant prior to questionnaire administration and MP sampling. Study data were managed in accordance with relevant guidelines and regulations (i.e., Regulation (EU) 2016/679 of the European Parliament and of the Council of 27 April 2016, on the protection of natural persons with regard to the processing of personal data and on the free movement of such data, and repealing Directive 95/46/EC (General Data Protection Regulation)). The study protocol was approved by the Independent Ethical Committee of Fondazione PTV – Policlinico Tor Vergata (Policlinico Tor Vergata Hospital – PTV, Rome 00,133 – see: http://www.ptvonline.it/cei_temp.asp), trial register number: 32.20.

### 2.2. Questionnaire Administration

The questionnaire included a general demographic section (gender, degree course, year of course, type of structure for the internship, and weekly attendance frequency) and a specific section, consisting of 7 items covering their habits related to the use of MPs (use in the healthcare environment, frequency of cleaning, method of cleaning, last cleaning date, use of a phone cover, use of the MPs with gloves, and means of transport used to reach university on the day of sampling). The seven items had a multiple-choice answer, with the possibility for some of them, to provide additional information. A space was also provided for any notes/suggestions the students wanted to add.

Enrollment sessions took place from October 2018 to February 2019 and were carried out at the Tor Vergata University, soon after classes. Some classes were attended by students from two or more degree courses. A maximum of 20 volunteers per session were enrolled. Each filled-out questionnaire was given a progressive number for data recording and processing.

### 2.3. Microbiological Analysis

After filling in the questionnaire, the students made their MPs available for microbiological analysis. Alginate-tipped sterile swabs were used to sample the touchscreen surface; the usual procedures for small environmental surfaces were applied [19,20,21,22,23,24]. Swabs were then re-suspended in 2.5 mL SRK solution (Copan Diagnostics, Murrieta, USA). Each swab tube was given the same progressive number as the questionnaire; the tubes were then immediately transferred to the Environmental Microbiology Laboratory of the University.

The subsequent analysis included a quantitative evaluation of the Heterotrophic Plate Count (HPC), both at 37 °C (HPC 37°C) and at 22 °C (HPC 22 °C), of Enterococci, of *E. coli* or total Coliforms and Staphylococci. In addition, identification of the isolated staphylococcal strains was performed, using biochemical tests.

The above microorganisms and parameters are commonly used in environmental analyses, as indicators of the hygienic quality of surfaces [24,25,26,27,28,29,30,31]. Moreover, Staphylococci, Enterococci, and Coliforms are often involved in HAIs [16,32,33] and were already isolated from MPs in previous studies [6,15,18,34,35].

The tubes were handled as follows:

- for the determination of HPC 37 °C and HPC 22 °C, 500 µL from each sample was poured in two empty sterile plates, then 16 mL of Plate Count Agar (PCA) medium (Biokar diagnostics, Allonne, France) was added and the plates were incubated at 37 °C and 22 °C, respectively, for 48/72 h;
- for the determination of Staphylococci, Enterococci, and *E. coli* or Coliforms, 250 µL per sample were spread on sterile plates containing the appropriate solidified media—Baird Parker Egg Yolk Tellurite Agar (Biokar diagnostics, France), Slanetz and Bartley agar (Biokar diagnostics, France), and Harlequin *E. coli* or Coliform chromogenic medium (Neogen Culture Media, Lansing, USA), respectively. The plates were then incubated at 37 °C for 48/72 h.

After the appropriate culture times, the plates were observed, and colony counts were carried out. The results were expressed as CFU/dm^2^ and recorded on a printed form.

For a qualitative evaluation of the staphylococcal species, the morphology of the colonies was evaluated macroscopically; then the different types were isolated and identified via the miniaturized biochemical test API Staph (Biomerieux, France), according to the manufacturer’s instructions.

### 2.4. Statistical Analysis

All data were recorded on an Excel^®^ worksheet. For descriptive analysis of the questionnaire, we expressed each variable in terms of number and percentage; regarding microbiological quantitative results, we expressed variables in terms of mean, standard deviation (SD), median, and interquartile range (IQR), stratifying according to questionnaire variables. For qualitative analysis, we built a Heatmap with R software v. 3.6.2 (The R Foundation, Vienna, Austria) [36]. We chose to categorize 3 of the continuous variables into dichotomous ones (i.e., HPC 37 °C and 22 °C and Staphylococci), using a threshold of 100 CFU/dm^2^. We applied a 100 CFU/dm^2^ threshold, according to Castiglia et al. [37], allowing us to highlight differences more effectively, across variables.

For the *Staphylococcus* species, *E. coli*, Coliforms, and Enterococci, we plotted the frequency of occurrence. In order to graphically show the relation between the questionnaire variables of interest and the bacterial concentrations, we plotted the samples in scatter plot, through R [36]. Bacterial concentrations were expressed as natural logarithm (ln) of CFU/dm^2^, while for each categorical variable the mean value and the 95% Confidence Interval (IC) were reported.

Quantitative analyses were performed through SPSS ver. 22.0 (SPSS Inc. Chicago, IL, USA). Kruskal-Wallis test was used to determine if there were differences in CFU/dm^2^ between groups, across the following variables—gender, year of the course, type of internship site, weekly attendance at the internship site, cleaning frequency, cleaning method, type of phone case, and means of transport used.

Variables with a significance threshold of *p* < 0.2 were used to run multiple linear regression models; Box-and-whisker plots were built to show the distributions of HPC 37 °C, HPC 22 °C, Enterococci, and Staphylococci for each variable selected for the multiple regression models.

## 3. Results

### 3.1. Demographic Description

Sampling was carried out in 11 enrollment sessions. The demographic characteristics of the population sample and the overall results of our survey are shown in Table 1. A total of 108 students agreed to take part in our study, accounting for about two-thirds of the total available population (*n* = 158). Most of them were female (85.5%), consistent with the overall population attending the considered degree courses in our university (data not shown). About half of the students (54.6%) were attending their training internship for 6 days a week, while the others (except for one of them) reported an attendance frequency of 5 days a week or less. The Nursing Course resulted the most represented degree course (54.6%), and the majority of students were attending their second bachelor year (64.8%).

About the touchscreen cleaning habits, up to 13.9% of the students declared to have never cleaned their phone, while only 2.8% of them stated to clean the phone daily. Most of the students (38.9%) used a disinfectant-based method of cleaning, including disinfectant gels, sprays, alcohol-based products, or a combination of these. The use of water-based products was the second most frequent method (29.6%), i.e., water and soap, or wet towels and wipes. Eighteen percent of the students used “dry” methods, such as glass-cleaning cloth, lens wipes, and handkerchiefs. With regard to the type of phone cover, most students (67.6%) had a silicone case, 11.1% of them had a flip-cover, while 21.3% did not use any cover at all.

Regarding the means of transport used to reach the University on the day of sampling, the answers were almost evenly split between public (39.8%) and private transport (45.4%).

The great majority (93.5%) of the enrolled students declared to use their phones while inside the hospital units, but 72.2% declared to never touch their MPs while wearing gloves; 14.8% actually used their MPs with gloves on, and only 2 (1.9%) changed the gloves soon afterwards.

### 3.2. Microbiological Results

All analyzed smartphones showed some degree of bacterial contamination, although with a wide variability on both quantitative and qualitative terms. Quantitative results of microbiological analyses for HPC at 37 °C and at 22 °C, *E. coli*, total Coliforms, Enterococci, and Staphylococci are reported in Supplementary material, Table S1.

HPC positivity was observed in 104 (96.3%) samples incubated at 37 °C and in 101 (93.5%) incubated at 22 °C. The four plates found to be negative for bacterial growth at 37 °C, showed some CFU at 22 °C; the opposite was noticed in the 7 samples found to be negative at 22 °C. Fourteen samples (13%) showed only HPC positivity but no Coliform, enterococcal, or staphylococcal growth. Total Coliforms were detected in 6.5% of the samples, Enterococci in 37.0%, while Staphylococci resulted highly represented (85.2%). *Escherichia coli* was never detected.

The most frequently isolated species of Staphylococci was *Staphylococcus epidermidis* (72.2% of the MPs), followed by *S. capitis* (13.9%), *S. saprophyticus* (5.6%), *S. warneri* (5.6%), *S. xylosus* (5.6%), *S. aureus* (3.7%), *S. chromogenes* (3.7%), *S. cohnii cohnii* (0.9%) and *S. simulans* (0.9%). *Micrococcus* spp., which was also detected by the API Staph test, was retrieved in 12.9% of the samples. A maximum of 3 different species were identified simultaneously in the same plate; such a situation occurred in 7 different samples; 31 MPs showed 2 different microbial species, while in the remaining 52, a single species was identified.

**Figure 1:**
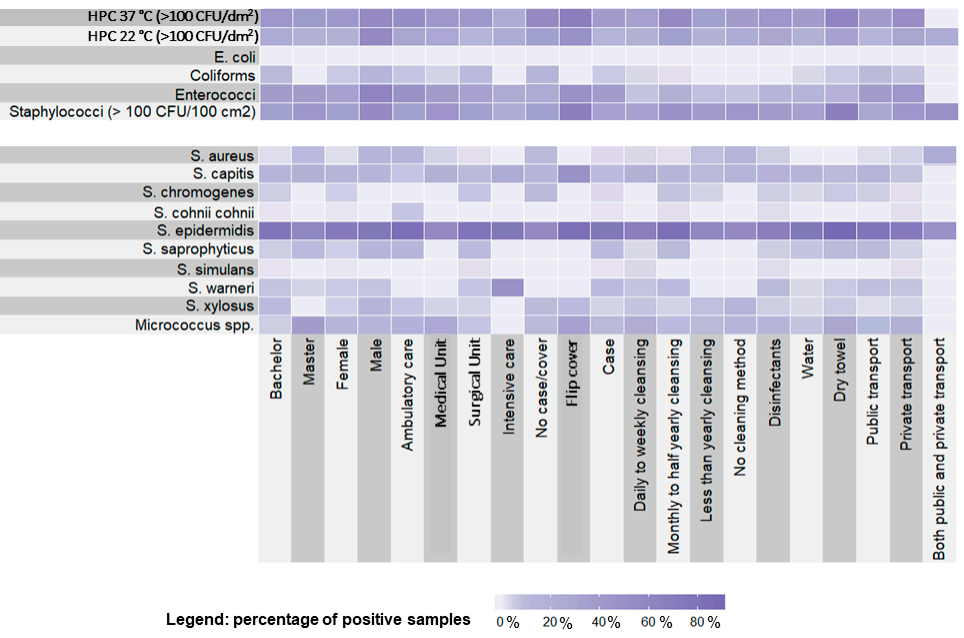
Heatmap showing the distribution of the microbiological findings in relation to students’ demographic characteristics and habits.

Figure 1 shows a Heatmap highlighting the quantitative presence of different bacterial strains, in relation to the described variables and at the two different incubation temperatures. In particular, HPC 37 °C and Staphylococci were well-represented in relation to all the tested variables, with higher loads in students using flip cover or dry towels. *S. epidermidis* was the more represented staphylococcal species in the tested samples, whereas *S. warneri* was found preferentially in Intensive Care Units and *S. capitis* was associated with the use of a flip cover.

**Figure 2:**
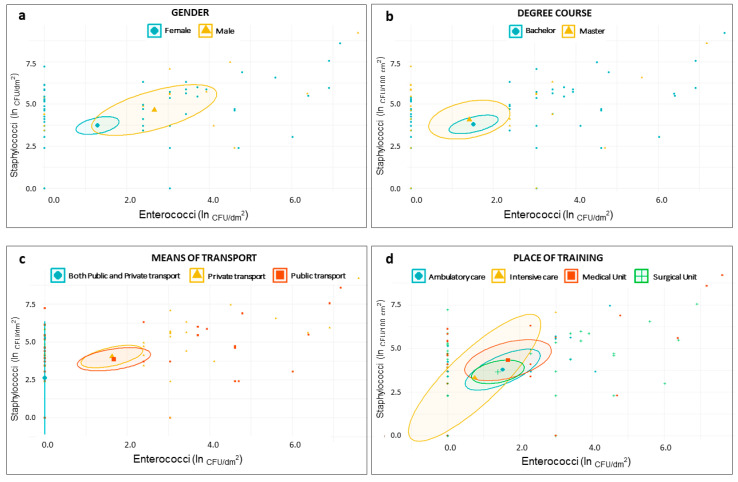
Scatter plots showing distribution of samples based on staphylococcal and enterococcal load—ln(CFU/dm^2^)—according to students’ demographic characteristics—gender (**a**); degree course (**b**); means of transport (**c**); and place of training (**d**).

**Figure 3:**
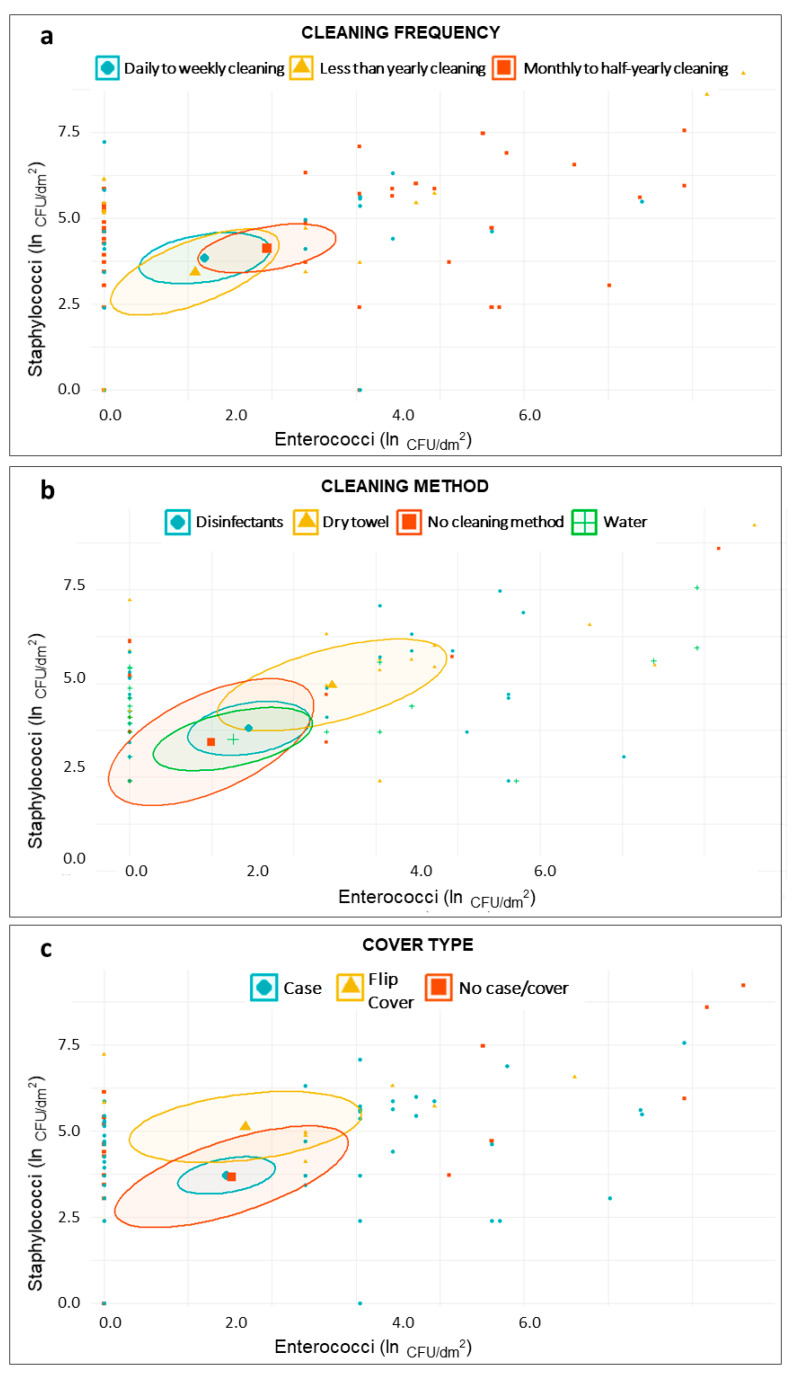
Scatter plots showing distribution of samples based on staphylococcal and enterococcal load—ln(CFU/dm^2^)—according to students’ smartphone-related habits—cleaning frequency (**a**); cleaning method (**b**); and cover type (**c**).

The scatter plots reported in Figure 2 and Figure 3 showed that the distribution of samples according to the staphylococcal and enterococcal loads was substantially overlapping when the samples were grouped according to the type of degree course (Master degree or Bachelor degree) (Figure 2b), the place of training (Figure 2d), and the cleaning frequency (Figure 3a). Comparatively lower loads were found in those who used both public and private transport (Figure 2c), although the low sample size (*n* = 4) was responsible for a wide confidence interval. On average, higher loads of Staphylococci and Enterococci were found in MPs from male individuals (Figure 2a) and in those using dry methods to clean the devices (Figure 3b); Staphylococci alone were present in greater quantities in individuals using flip covers (Figure 3c). A scatter plot on *E. coli* and Coliforms was not built, due to their rare detection in our samples.

**Figure 4:**
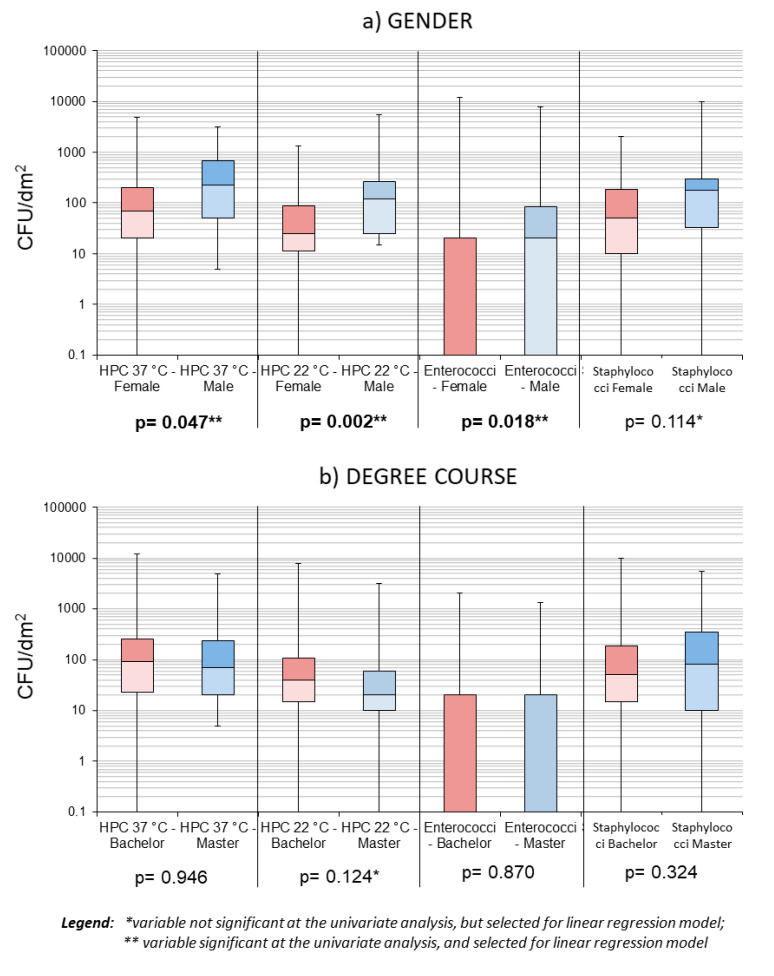
Box plots showing samples’ distribution by gender (**a**) and level of study (**b**).

**Figure 5:**
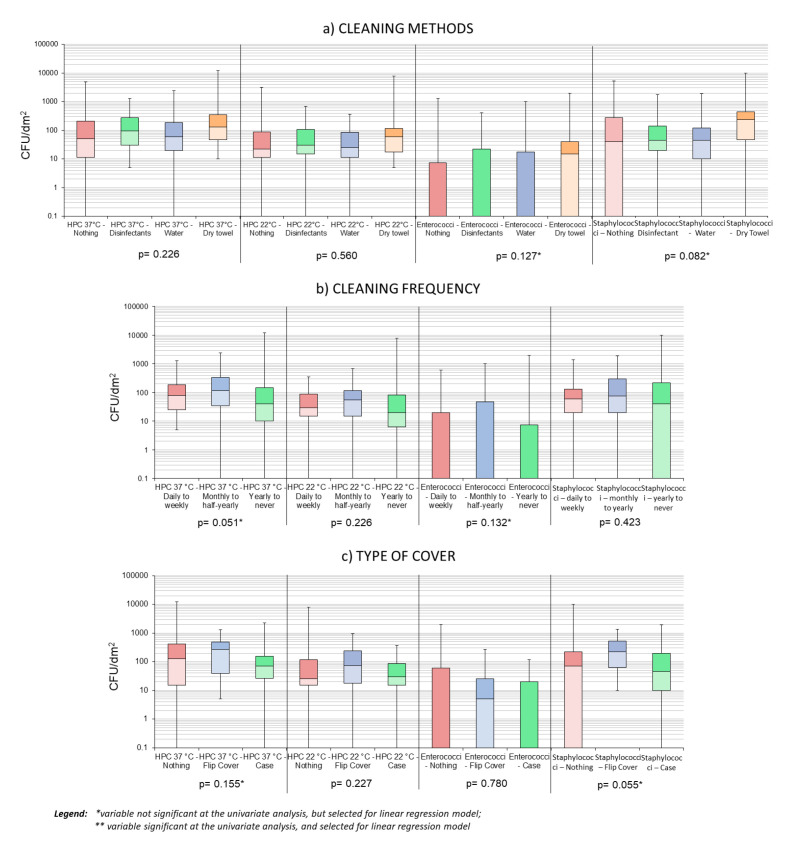
Box plots showing samples’ distribution by cleaning method (**a**), cleaning frequency (**b**), and type of cover used (**c**).

At the univariate analysis level, only gender was found to significantly influence bacterial loads, in terms of HPC 37 °C, HPC 22 °C, and Staphylococci (*p* < 0.05); using significance threshold of *p* < 0.2, to search for variables to inform regression models, we found more variables to be included in the models (see Figure 4 and Figure 5).

Regarding Coliforms, only one predictor was identified (i.e., the cycle of studies), and the consequent linear regression model resulted in an R = 0.107 (see also Table S2 in supplementary material). Despite the selection of several variables, no model could adequately predict the variables of interest—the type of cover, gender, frequency, and method of cleaning combined, could only explain 17% of the HPC 37 °C variability, while the type of cover, method of cleaning, and gender combined explained about 16% of the staphylococcal load variability. The remaining models all had R-square < 0.15.

## 4. Discussion

The results of our study showed that the touchscreens of MPs were often colonized by bacteria, including pathogenic and opportunistic species, in line with previous studies on this subject [1,2,3,5,6,7,8,9,12,13,14,15,16,18,19,35,38,39,40].

All MPs in our sample showed at least some degree of bacterial contamination, different from other previous reports [1,3,5,6,7,8,9,12,13,14,15,16,19,35,38,39,40], but in accordance with some other ones [2,18]. This difference could be explained by the technique we adopted, namely standard methods of environmental microbiology, evaluating mesophilic and psychrophilic flora, through the HPC 37 °C and HPC 22 °C [25,26,28,41,42]. Even if HPC was a generic value per se, it was considered a good indicator of hygiene quality of surfaces, regardless of specific bacterial species that could be identified [26]. However, in our study, the usual HPC threshold values of <500 CFU/dm^2^ [26,30] or <250 CFU/dm^2^ [25,28,41] did not seem to be sufficient to guarantee adequate hygienic quality level of the MPs surfaces. Based on our results, two different tolerance thresholds for HPC 37 °C could be proposed, depending on the risk level of the hospital unit—a restrictive cut-off of 15 CFU/dm^2^ for units at high risk for HAIs (only staphylococcal presence in 44% of MPs, in the absence of *S. aureus* in our sample), as already at 20 CFU/dm^2^ of HPC 37 °C, Enterococci were observed in 6% of samples and Staphylococci in 56%; and a higher threshold of 35 CFU/dm^2^ was observed for the other units (0% Coliforms, 8% Enterococci, but with loads <100 CFU/dm^2^, and 60% Staphylococci, loads <100 CFU/dm^2^ in our sample). With higher values of HPC 37 °C, we first observed the occurrence of high staphylococcal loads (230 CFU/dm^2^ for HPC 37 °C of 40 CFU/dm^2^ ), then higher enterococcal loads (100 CFU/dm^2^ for HPC 37 °C of 45 CFU/dm^2^), and eventually the appearance of Coliforms (for HPC 37 °C of 65 CFU/dm^2^) and of *S. aureus* (for HPC 37 °C of 70 CFU/dm^2^).

The presence of Coliforms and Enterococci indicates bad individual hygienic habits or accidental contamination through the fecal route [26,30,43]. *S. aureus* was considered to be a marker of insufficient hygienic quality for surfaces in hospital settings [25,26]; its pathogenicity and widespread antibiotic resistances must be also taken into account [44,45].

Our findings showed that male gender, flip cover, and dry towel were predictors of the presence of HPC 37 °C, Enterococci and Staphylococci. All measured microbiological parameters were significantly higher in the smartphones of male participants. According to previous studies, female students reported to have better attitudes towards hand hygiene, and a higher rate of hand hygiene practice than the males [46,47]. The report of higher bacterial loads on MPs owned by male individuals was inconsistent in the literature [9,48], and sometimes a better attitude towards hand hygiene was associated with worse hand hygiene practice [49,50].

As expected, coagulase-negative Staphylococci (CoNS) were the most commonly isolated bacteria from our samples, being part of the human skin flora [51], as documented by the many species identified, namely *S. epidermidis*, *S. capitis*, *S. saprophyticus*, *S. warneri*, *S. xylosus*, *S. chromogenes*, *S. cohnii cohnii*, and *S. simulans*.

In our series, the presence of *S. aureus* colonies was observed in a much smaller percentage of cases (3.7%), when compared with the presence of CoNS (85/108; 78.7%). This result was in line with previous observations on the subject [1,3,5,6,7,8,12,13,15,16,18,30,38,40,52], although our *S. aureus* values were even lower than those reported in most of such studies.

To date, the etiological role of CoNS in human diseases was extensively reported, especially in hospital settings, where some of these species might act as leading opportunistic pathogens or could even have emerged as truly pathogenic bacteria, particularly in immunocompromised patients [32,52].

In addition, these bacteria showed increasing tendency to develop antibiotic resistances, making the management of such infections even more difficult [32,53,54].

*Staphylococcus epidermidis* was the most frequently isolated staphylococcal species from our samples. It possessed a high capacity of biofilm production, allowing it to adhere to and survive on many different biological or artificial surfaces [55]. For this reason, it was considered to be the main cause of implanted medical device contamination, and thus of infection of patients carrying such devices [56]. In our series, *S. epidermidis* was found in 78 out of 108 of the samples, representing 72.2% of the overall population and 85.7% of all plates that were positive for the *Staphylococcal* species. It could be inferred that the biofilm-producing features of this species could make it highly resistant on touchscreen surfaces, allowing it to be easily carried throughout the facilities/hospital units. Therefore, it could be useful to adopt appropriate disinfectant cleaning methods for the decontamination of MPs.

*Staphylococcus saprophyticus*, although being a commensal species in the human skin and gastrointestinal tract, was associated with lower urinary tract infections (UTIs)—in particular, it was reported as being second only to *Escherichia coli* as the etiological agent of UTIs in young and middle-aged women [32,57]. Moreover, similar to *S. epidermidis*, it might become highly pathogenic when reaching the bloodstream, through intravenous drug administration, dialysis, catheter insertions, or spinal anesthesia [3].

*S. capitis* is a part of the human cutaneous bacterial flora, especially on the scalp and arms. It is an opportunistic pathogen, causing prosthetic joints infections and cases of bloodstream infections from catheters, bacterial endocarditis, peritonitis in CAPD, and neonatal sepsis. Thus, it is considered to be a serious pathogen, especially in neonatal settings [32,54].

Micrococci are nowadays reclassified under the class Actinobacteria, [32] thus, phylogenetically distinct from Staphylococci; nevertheless, many species of the two genera share the same microenvironments in the human body [58]. In addition, just like some *Staphylococcus* species, Micrococci can be found on food, as well as in the environment. Micrococcal species were reported to cause pulmonary infections, bacterial endocarditis, and bloodstream infections from catheters; the risk seemed to be increased in immunocompromised patients [58,59].

Colonization of MPs by *Coliform* and *Enterococcal* species is very important from a clinical point of view; not only are they a well-known cause of HAIs, but they also show a wide degree of emerging antibiotic resistances, thus, representing a life-threatening danger, especially in vulnerable patients (i.e., elderly, immunosuppressed, and those with multiple comorbidities) [33,60,61].

In our sample, cellular phones that were frequently cleaned as well as those that were seldom or never cleaned, showed lower contamination levels than the MPs in the intermediate cleaning frequencies. For explaining this finding, we could assume the possibility of the development, over time, of a local flora on the MPs’ screens, counteracting an excessive proliferation of a single bacterial species through competitive inhibition. In this setting, therefore, we could formulate the hypothesis that cleaning the touchscreen could alter the established microbial balance, similar to what happens for hand microbiota [62], giving rise to a greater and disordered bacterial growth after cleaning, once the effect of the disinfectant fades off.

On the other hand, as already shown in previous works, the bacterial contamination of an MP could simply reflect the particular skin bacterial flora (microbiota) of an individual [51,63]. The microbiota hypothesis could represent one of the possible explanations to our results—in fact, no significant differences in bacterial contamination were observed in relation to the degree course attended, the type of transport used (private or public), and the department of training. In this sense, especially in a healthcare environment, it would become more appropriate than ever to focus on proper hygienic procedures (hand washing, proper use of disposable gloves), before and after performing activities on patients, and possibly on cleaning MPs, at least before and after hospital working shifts.

In addition, this underlined the importance of accurate hand hygiene even outside the working environment, as emphasized by the WHO [26]; in fact, the risk of transferring pathogenic and possibly drug-resistant bacteria from the hospital to the community, and not only the opposite, should be considered as well [64].

Our findings about phone usage by students were very similar to those of a survey conducted in North America at the onset of Covid-19 pandemic, which aimed to find out the habits of nurses on MPs use in different hospital settings (our results compared to the PDI healthcare study’ ones—use of mobile phones in hospital: 93% vs. 84%; use without gloves on—72% vs. 84%; frequent cleaning of the MPs—33% vs. 41%); the only relevant exception was the use of disinfectants for MPs cleaning, which we found to be less frequent (39% vs. 72%) [65]. These data could be seen as an opportunity to improve knowledge and practices on MPs management in healthcare settings.

## 5. Conclusions

This study allowed us to assess the contamination of MPs in terms of microbiological indicators and to correlate it to the characteristics and habits of the examined population in a healthcare setting. This experience also gave us the opportunity to inform the students about the microbiological risks their MPs can pose, thus, taking a first step towards a more systematic educational intervention on the potential risks related to these devices, in healthcare degree courses. Training students of healthcare professions about the possible risks associated with the use of MPs in the field of HAIs, could be an example of good practice to be implemented into University degree courses.

In accordance to what was reported by Raza et al., 2017 [3], it is neither advisable nor useful to ban the use of mobile phones in healthcare settings, as MPs proved to be highly valuable for rapid communication between health professionals, patients, and for ad-hoc applications. Thus, the future objectives should be focused on the development of specific guidelines for the proper management of these devices (e.g., frequency and method of decontamination, proper use of gloves, etc.) in hospital settings [66,67], just like other usual medical tools.

Particular habits, such as the use of a flip cover and cleaning the MP with a dry towel, in our work were associated with higher HPC 37 °C, staphylococcal, and enterococcal growth—in our opinion, if these results were confirmed through further research, these habits should be discouraged among HCWs.

Still, the importance of proper hand hygiene should never be underestimated. MPs are one of the most highly touched surfaces according to the CDCs, and, during the current Covid-19 pandemic, hand hygiene has been recommended as a key infection control strategy by all leading health societies [19,68]; besides, it has long been established as the main standard precaution for the prevention of HAIs [3]. MPs are a high-risk surface, as they can come into contact with the hands, face, mouth, and droplets, with the potential effect of negating hand hygiene, and to act as Trojan horses [19,68].

The actual increased societal awareness has led major MPs companies such as Apple, Samsung, and Google to release guidance for proper MPs disinfection [65], while CDCs recently published advices to be followed for cleaning and disinfecting high touch surfaces like MPs, at home [68]; according to CDCs, when no producer’s guidance is available, alcohol-based wipes or sprays containing at least 70% alcohol should be used to sanitize electronic devices.

To the best of our knowledge, only two other studies on bacterial contamination of MPs were performed in Italy so far [15,69].

This work was a preliminary, hygienic-sanitary-oriented, evaluation of bacterial colonization of MPs of healthcare professions students, performed in a territorially limited setting.

Further, larger studies are planned to be conducted in the future, extending the focus on other microorganisms responsible for HAIs, or evaluating the antibiotic resistances of the retrieved species.

## Figures and Tables

**Table 1:** Demographic characteristics and habits of the sampled students.

Variable	Values	*n*	%
Gender *	Male	16	14.8
	Female	92	85.2
Course of study	Nursing sciences	59	54.6
	Obstetrics	29	26.9
	Hearing aid techniques	3	2.8
	Master in management for coordination of health professions	16	14.8
	Unknown	1	0.9
Year of the course *	First year, bachelor course	0	0
	Second year, bachelor course	70	64.8
	Third year, bachelor course	11	10.2
	First year, master course	26	24.1
	Second year, master course	1	0.9
Type of internship site *	Ambulatory care	17	15.7
	Medical ward	26	24.1
	Surgical ward	51	47.2
	Intensive care	4	3.7
	Not reported	10	9.3
Weekly attendance at the internship site *	7 days	1	0.9
	6 days	59	54.6
	5 days	19	17.6
	4 days	5	4.6
	3 days	16	14.8
	<3 days	5	4.6
	Not reported	3	2.8
Cleaning frequency *	Daily	3	2.8
	Weekly	29	26.9
	Monthly	28	25.9
	Half-yearly	20	18.5
	Yearly	13	12.0
	Never	15	13.9
Cleaning method *	Disinfectants	42	38.9
	Water	32	29.6
	Dry towel	18	16.7
	Nothing	16	14.8
Last cleaning performed	One day to a week before sampling	36	33.3
	Two weeks to six months before sampling	41	38.0
	Never	18	16.7
	Not reported	13	12.0
Type of phone case *	Flip cover	12	11.1
	Case	73	67.6
	No case/cover	23	21.3
Means of transport used *	Public	43	39.8
	Private	49	45.4
	Both public and private	4	3.7
	Not reported	12	11.1
Use of the smartphone during training in hospital	Yes	101	93.5
	No	7	6.5
Use of the smartphone with gloves during training	Yes	16	14.8
	Yes, but then I change the gloves	2	1.9
	No	78	72.2
	Not reported	12	11.1

* Variables selected for statistical analyses.

## Supplementary Materials

The following are available online at https://www.mdpi.com/2076-2607/8/7/1075/s1. Table S1: Microbiological quantitative results in relation to the main parameters of interest. Table S2: Multiple linear regression models.

## References

[1] Akinyemi K.O., Atapu A.D., Adetona O.O., Coker A.O. (2009). The potential role of mobile phones in the spread of bacterial infections. J. Infect. Dev. Countr..

[2] Kirkby S., Biggs C. (2016). Cell Phones in the Neonatal Intensive Care Unit: How to Eliminate Unwanted Germs. Adv. Neonatal Care.

[3] Raza I., Raza A., Razaa S.A., Sadar A.B., Qureshi A.U., Talib U., Chi G. (2017). Surface Microbiology of Smartphone Screen Protectors Among Healthcare Professionals. Cureus.

[4] Rahi P., Kurli R., Khairnar M., Jagtap S., Pansare A.N., Dastager S.G., Shouche Y.S. (2017). Description of Lysinibacillus telephonicus sp. nov., isolated from the screen of a cellular phone. Int. J. Syst. Evol. Microbiol..

[5] Kotris I., Drenjančević D., Talapko J., Bukovski S. (2017). Identification of microorganisms on mobile phones of intensive care unit health care workers and medical students in the tertiary hospital. Med. Glas. (Zenica).

[6] Brady R.R., Wasson A., Stirling I., McAllister C., Damani N.N. (2006). Is your phone bugged? The incidence of bacteria known to cause nosocomial infection on healthcare workers’ mobile phones. J. Hosp. Infect..

[7] Karkee P., Madhup S.K., Humagain P., Thaku N., Timilsina B. (2017). Mobile phone: A possible vector of bacterial transmission in hospital setting. Kathmandu Univ. Med. J..

[8] Chang C.H., Chen S.Y., Lu J.J., Chang C.J., Chang Y., Hsieh P.H. (2017). Nasal colonization and bacterial contamination of mobile phones carried by medical staff in the operating room. PLoS ONE.

[9] Bhoonderowa A., Gookool S., Biranjia-Hurdoyal S.D. (2014). The importance of mobile phones in the possible transmission of bacterial infections in the community. J. Community Health.

[10] Verran J. (2012). The microbial contamination of mobile communication devices. J. Microbiol. Biol. Educ..

[11] Aronson S.H. (1977). The Lancet on the telephone 1876–1975. Med. Hist..

[12] Kumar B.V., Hobani Y.H., Abdulhaq A., Jerah A.A., Hakami O.M., Eltigani M., Bidwai A.K. (2014). Prevalence of antibacterial resistant bacterial contaminants from mobile phones of hospital inpatients. Libyan J. Med..

[13] Datta P., Rani H., Chander J., Gupta V. (2009). Bacterial contamination of mobile phones of health care workers. Indian J. Med. Microbiol..

[14] Gallegos C., McDuffee V., Hong-Engelhard C., Boeck C. (2018). Hold the phone: Mobilizing against cell phone pathogens. Nursing.

[15] Di Lodovico S., Del Vecchio A., Cataldi V., Di Campli E., Di Bartolomeo S., Cellini L., Di Giulio M. (2018). Microbial Contamination of Smartphone Touchscreens of Italian University Students. Curr. Microbiol..

[16] Nwankwo E.O., Ekwunife N., Mofolorunsho K.C. (2014). Nosocomial pathogens associated with the mobile phones of healthcare workers in a hospital in Anyigba, Kogi state, Nigeria. J. Epidemiol. Glob. Health.

[17] Ulger F., Dilek A., Esen S., Sunbul M., Leblebicioglu H. (2015). Are healthcare workers’ mobile phones a potential source of nosocomial infections? Review of the literature. J. Infect. Dev. Countr..

[18] Beckstrom A.C., Cleman P.E., Cassis-Ghavami F.L., Kamitsuka M.D. (2013). Surveillance study of bacterial contamination of the parent’s cell phone in the NICU and the effectiveness of an anti-microbial gel in reducing transmission to the hands. J. Perinatol..

[19] Olsen M., Campos M., Lohning A., Jones P., Legget J., Bannach-Brown A., McKirdy S., Alghafri R., Tajouri L. (2020). Mobile phones represent a pathway for microbial transmission: A scoping review. Travel. Med. Infect. Dis..

[20] CDC (Centers for Disease Control and Prevention) (2012). The National Institute for Occupational Safety and Health (NIOSH). Surface Sampling Procedures for *Bacillus anthracis* Spores from Smooth, Non-Porous Surfaces. https://www.cdc.gov/niosh/topics/emres/surface-sampling-bacillus-anthracis.html.

[21] Brown G.S., Betty R.G., Brockmann J.E., Lucero D.A., Souza C.A., Walsh K.S., Boucher R.M., Tezak M.S., Wilson M.C., Rudolph T. (2007). Evaluation of rayon swab surface sample collection method for Bacillus spores from non porous surfaces. J. Appl. Microbiol..

[22] Sanderson W.T., Hein M.J., Taylor L., Curwin B.D., Kinnes G.M., Seitz T.A., Popovic T., Holmes H.T., Kellum M.E., McAllister S.K. (2002). Surface sampling methods for Bacillus anthracis spore contamination. Emerg. Infect. Dis..

[23] International Organization for Standardization (2004). Microbiology of Food and Animal Feeding Stuffs—Horizontal Methods for Sampling Techniques from Surfaces Using Contact Plates and Swabs.

[24] De Filippis P., Mozzetti C., Messina A., D’Alò G.L. (2018). Prevalence of Legionella in retirement homes and group homes water distribution systems. Sci. Total Environ..

[25] Amodio E., Cannova L., Villafrate M.R., Merendino A.M., Aprea L., Calamusa G. (2014). Analytical performance issues: Comparison of ATP bioluminescence and aerobic bacterial count for evaluating surface cleanliness in an Italian hospital. J. Occup. Environ. Hyg..

[26] Dancer S.J. (2004). How do we assess hospital cleaning? A proposal for microbiological standards for surface hygiene in hospitals. J. Hosp. Infect..

[27] Edberg S.C., Rice E.W., Karlin R.J., Allen M.J. (2000). *Escherichia coli*: The best biological drinking water indicator for public health protection. Symp. Ser. Soc. Appl. Microbiol..

[28] Griffith C.J., Cooper R.A., Gilmore J., Davies C., Lewis M. (2000). An evaluation of hospital cleaning regimes and standards. J. Hosp. Infect..

[29] Huang P.Y., Shi Z.Y., Chen C.H., Den E., Huang H.M., Tsai J.J. (2013). Airborne and surface-bound microbial contamination in two Intensive care units of a medical center in Central Taiwan. Aerosol Air Qual. Res..

[30] Malik R.E., Cooper R.A., Griffith C.J. (2003). Use of audit tools to evaluate the efficacy of cleaning systems in hospitals. Am. J. Infect. Control..

[31] World Health Organization, WHO Patient Safety (2009). WHO Guidelines on Hand Hygiene in Health Care: A Summary.

[32] Becker K., Heilmann C., Peters G. (2014). Coagulase-negative staphylococci. Clin. Microbiol. Rev..

[33] McDanel J., Schweizer M., Crabb V., Nelson R., Samore M., Khader K., Blevins A.E., Diekema D., Chiang H.Y., Nair R. (2017). Incidence of Extended-Spectrum β-Lactamase (ESBL)-Producing Escherichia coli and Klebsiella Infections in the United States: A Systematic Literature Review. Infect. Control Hosp. Epidemiol..

[34] Chao Foong Y., Green M., Zargari A., Siddique R., Tan V., Brain T., Ogden K. (2015). Mobile Phones as a Potential Vehicle of Infection in a Hospital Setting. J. Occup. Environ. Hyg..

[35] Ustun C., Cihangiroglu M. (2012). Health care workers’ mobile phones: A potential cause of microbial cross-contamination between hospitals and community. J. Occup. Environ. Hyg..

[36] The R Foundation The R Project for Statistical Computing. https://www.r-project.org/.

[37] Castiglia P., Liguori G., Montagna M.T., Napoli C., Pasquarella C., Bergomi M., Fabiani L., Monarca S., Petti S., Siti Working Group Hygiene in Dentistry (2008). Italian multi-center study on infection hazards during dental practice: Control of environmental microbial contamination in public dental surgeries. BMC Public Health.

[38] Brady R.R., Fraser S.F., Dunlop M.G., Paterson-Brown S., Gibb A.P. (2007). Bacterial contamination of mobile communication devices in the operative environment. J. Hosp. Infect..

[39] Jeske H.C., Tiefenthaler W., Hohlrieder M., Hinterberger G., Benzer A. (2007). Bacterial contamination of anaesthetists’ hands by personal mobile phone and fixed phone use in the operating theatre. Anaesthesia.

[40] Brady R.R., Hunt A.C., Visvanathan A., Rodrigues M.A., Graham C., Rae C., Kalima P., Paterson H.M., Gibb A.P. (2011). Mobile phone technology and hospitalized patients: A cross-sectional surveillance study of bacterial colonization, and patient opinions and behaviours. Clin. Microbiol. Infect..

[41] Sherlock O., O’Connell N., Creamer E., Humphreys H. (2009). Is it really clean? An evaluation of the efficacy of four methods for determining hospital cleanliness. J. Hosp. Infect..

[42] De Filippis P., Mozzetti C., Messina A., D’Alò G. (2018). Data on Legionella prevalence and water quality in showers of retirement homes and group homes in the Province of Rome, Lazio Region, Italy. Data Brief.

[43] Chiller K., Selkin B.A., Murakawa G.J. (2001). Skin microflora and bacterial infections of the skin. J. Investig. Dermatol. Symp. Proc..

[44] Walvick M.D., Amato M. (2011). Ophthalmic methicillin-resistant Staphylococcus aureus infections: Sensitivity and resistance profiles of 234 isolates. J. Community Health.

[45] Galindo G.R., Casey A.J., Yeung A., Weiss D., Marx M.A. (2012). Community associated methicillin resistant Staphylococcus aureus among New York City men who have sex with men: Qualitative research findings and implications for public health practice. J. Community Health.

[46] Anderson J.L., Warren C.A., Perez E., Louis R.I., Phillips S., Wheeler J., Cole M., Misra R. (2008). Gender and ethnic differences in hand hygiene practices among college students. Am. J. Infect. Control..

[47] Kinnison A., Cottrell R.R., King K.A. (2004). Proper hand-washing techniques in public restrooms: Differences in gender, race, signage, and time of day. Am. J. Health Educ..

[48] Koroglu M., Gunal S., Yildiz F., Savas M., Ozer A., Altindis M. (2015). Comparison of keypads and touch-screen mobile phones/devices as potential risk for microbial contamination. J. Infect. Dev. Ctries.

[49] Cruz J.P., Cruz C.P., Al-Otaibi A.S.D. (2015). Gender differences in hand hygiene among Saudi nursing students. Int. J. Infect. Control.

[50] Cruz J.P., Bashtawi M.A. (2016). Predictors of hand hygiene practice among Saudi nursing students: A cross-sectional self-reported study. J. Infect. Public Health.

[51] Grice E.A., Segre J.A. (2011). The skin microbiome. Nat. Rev. Microbiol..

[52] Argemi X., Hansmann Y., Riegel P., Prévost G. (2017). Is Staphylococcus lugdunensis Significant in Clinical Samples?. J. Clin. Microbiol..

[53] Vestergaard M., Frees D., Ingmer H. (2019). Antibiotic Resistance and the MRSA Problem. Microbiol. Spectr..

[54] Argemi X., Hansmann Y., Prola K., Prévost G. (2019). Coagulase-Negative Staphylococci Pathogenomics. Int. J. Mol. Sci..

[55] Gomes F., Teixeira P., Oliveira R. (2014). Mini-review: Staphylococcus epidermidis as the most frequent cause of nosocomial infections: Old and new fighting strategies. Biofouling.

[56] Fey P.D., Olson M.E. (2010). Current concepts in biofilm formation of Staphylococcus epidermidis. Future Microbiol..

[57] Widerström M., Wiström J., Ferry S., Karlsson C., Monsen T. (2007). Molecular epidemiology of Staphylococcus saprophyticus isolated from women with uncomplicated community-acquired urinary tract infection. J. Clin. Microbiol..

[58] Smith K.J., Neafie R., Yeager J., Skelton H.G. (1999). Micrococcus folliculitis in HIV-1 disease. Br. J. Dermatol..

[59] Valdivia-Arenas M.A., Sood N. (2008). Micrococcus bloodstream infection in patients with pulmonary hypertension on epoprostenol. Infect. Dis. Clin. Pract..

[60] Weiner L.M., Webb A.K., Limbago B., Dudeck M.A., Patel J., Kallen A.J., Edwards J.R., Sievert D.M. (2016). Antimicrobial-Resistant Pathogens Associated With Healthcare-Associated Infections: Summary of Data Reported to the National Healthcare Safety Network at the Centers for Disease Control and Prevention, 2011–2014. Infect. Control Hosp. Epidemiol..

[61] Perez F., Van Duin D. (2013). Carbapenem-resistant Enterobacteriaceae: A menace to our most vulnerable patients. Cleve Clin. J. Med..

[62] Fierer N., Hamady M., Lauber C.L., Knight R. (2008). The influence of sex, handedness, and washing on the diversity of hand surface bacteria. Proc. Natl. Acad. Sci. USA.

[63] Meadow J.F., Altrichter A.E., Green J.L. (2014). Mobile phones carry the personal microbiome of their owners. PeerJ.

[64] Jiang L., Ng I.H.L., Hou Y., Li D., Tan L.W.L., Ho H.J.A., Chen M.I.C. (2018). Infectious disease transmission: Survey of contacts between hospital-based healthcare workers and working adults from the general population. J. Hosp. Infect..

[65] PDI Healthcare 9 Stats on Cell Phone Cleaning in Hospitals: Results from Our Survey of 100 Nurses. Posted May 20, 2020. https://pdihc.com/blog/9-stats-on-cell-phone-cleaning-in-hospitals-results-from-our-survey-of-100-nurses/.

[66] Brady R.R., Verran J., Damani N.N., Gibb A.P. (2009). Review of mobile communication devices as potential reservoirs of nosocomial pathogens. J. Hosp. Infect..

[67] Visvanathan A., Rodrigues M.A., Brady R., Gibb A.P. (2012). Mobile phone usage in the clinical setting: Evidence-based guidelines for all users is urgently required. Am. J. Infect. Control.

[68] Centers for Disease Control and Prevention (CDC) (2020). Cleaning and Disinfecting Your Home. Page Last Reviewed: May 27, 2020. https://www.cdc.gov/coronavirus/2019-ncov/prevent-getting-sick/disinfecting-your-home.html.

[69] Orsi G.B., Natale F., d’Ettorre G., Protano C., Vullo V., De Curtis M. (2015). Mobile phone microbial contamination among neonatal unit healthcare workers. Infect. Control Hosp. Epidemiol..

[70] Cicciarella Modica D., D’Alò G.L., Mozzetti C., Messina A., Maurici M., Pica F., De Filippis P. (2019). “Studio sulla contaminazione batterica degli smartphone di studenti iscritti a corsi di laurea delle professioni sanitarie presso l’Università degli Studi di Roma ‘Tor Vergata’: Risultati preliminari”, in: Atti Del 52° Congresso Nazionale: Società Italiana Di Igiene, Medicina Preventiva E Sanità Pubblica (SItI). J. Prev. Med. Hyg..

