# Supplementary material for: Taking Screenshots of the Invisible: A Study on Bacterial Contamination of Mobile Phones from University Students of Healthcare Professions in Rome, Italy
Source: Microorganisms. 2020 Jul 19;8(7):1075. doi: 10.3390/microorganisms8071075 (PMC7409191; doi:10.3390/microorganisms8071075)
Supplement: Supplementary file 1 [file microorganisms-08-01075-s001.pdf]

Cicciarella Modica D, Maurici M, D'Alò GL, Mozzetti C, Messina A, Distefano A, Pica F, De Filippis P. **Taking screenshots of the invisible: a study on bacterial contamination of mobile phones from university students of healthcare professions in Rome, Italy.** *Microorganisms*.

SUPPLEMENTARY MATERIAL

Index:

Page 2: **Table S1**. Microbiologic quantitative results in relation to the main parameters of interest.

Page 3: **Table S2**. Multiple linear regression models

Table S1. Microbiologic quantitative results in relation to the main parameters of interest.

		HPC 37 °C, CFU/dm^2		HPC 22 °C, CFU/ dm^2		Coliforms, CFU/ dm^2		Enterococci, CFU/dm^2		Staphylococci, CFU/dm^2	
		Mean; SD	Median; IQR	Mean; SD	Median; IQR	Mean; SD	Median; IQR	Mean; SD	Median; IQR	Mean; SD	Median; IQR
**Total**		361.9; 1285.1	82.5; 23.8-235.0	180.6; 817.6	30.0; 15.0 -100.0	2.9; 15.5	0; 0-0	78.3; 276.3	0; 0-20.0	314.6; 1112.1	60.0; 10.0-222.5
**Gender**	Female	241.2; 619.6	70.0; 20-202.5	99.2; 345.4	25; 11.3-88.8	2.8; 16.3	0; 0-0	59.6; 211.0	0; 0-20	210.9; 612.3	50; 10-187.5
	Male	1055.6; 2974.6	222.5; 50-670	649.1; 1941.7	117.5; 25-267.5	3.1; 10.1	0; 0-0	186.3; 509.8	20; 0-82.5	911.3; 2470.1	175; 32.5-295
**Year of training**	Bachelor	361.2; 1385.9	90; 22.5-255	172.5; 875.2	40; 15-105	3.8; 17.9	0; 0-0	81.9; 284.7	0; 0-20	284.4; 1141.1	50; 15-185
	Master	363.9; 941.2	70; 20-230	205; 627.3	20; 10-60	0; 0	0; 0-0	67.8; 254.2	0; 0-20	405.2; 1035.2	80; 10-350
**Degree course**	Nursing sciences	523.9; 1699.9	95; 30-340	258.6; 1092.8	30; 15-115	2.5; 14	0; 0-0	110.3; 347.3	0; 0-20	420; 1463.1	60; 20-240
	Obstetrics	182.9; 423.7	75; 20-150	52.4; 69,6	30; 12.5-72.5	5.5; 22.5	0; 0-0	45.9; 184.7	0; 0-20	170.7; 359.4	50; 10-205
	Audio-prosthetics	373.3; 311.5	300; 105-nd	316.7; 328.4	150; 105-ND	0; 0	0; 0-0	53.3; 40.4	60; 10-ND	640; 953.9	140; 40-ND
	Management Master	103.1; 130.1	52.5; 16.3-181.3	108.8; 248.1	17.5; 5-58.8	0; 0	0; 0-0	28.8; 70	0; 0-20	142.5; 201.8	60; 10-242.5
**Place of Training**	Ambulatory care	174.4; 204.5	105; 22.5-275	105.9; 174.7	55; 10-110	7.1; 29.1	0; 0-0	15.9; 25.3	0; 0-25	189.4; 412.3	40; 10-215
	Medical ward	799.2; 2498.5	82.5; 20-322.5	507.9; 1635.2	35; 15-115	1.5; 7.8	0; 0-0	162.3; 468.5	0; 0-20	758.5; 2152.7	80; 27.5-367.5
	Surgical ward	185.7; 358.4	70; 35-150	62.1; 83.6	30; 15-90	1; 3.6	0; 0-0	56.1; 172	0; 0-20	162.2; 333.7	50; 10-180
	Intensive care	213.8; 404.2	15; 6.3-620	72.5; 135.1	7.5; 1.3-208.8	0; 0	0; 0-0	5; 10	0; 0-15	310; 580.4	30; 2.5-897.5
**Weekly Attendance**	≤3 days	140.7; 188.5	70; 20-155	102.1; 161.7	45; 15-105	0; 0	0; 0-0	18.1; 31.6	0; 0-20	154.3; 372.6	50; 10-140
	4-5 days	854.8; 2598.2	67.5; 20-266.3	534.4; 1702.7	15; 10-73.8	6.7; 25.5	0; 0-0	142.1; 480.7	0; 0-2.5	850.8; 2240.4	60; 20-375
	6-7 days	236.5; 460.1	90; 30-216.3	68.3; 82.8	35; 20-91.3	2.5; 13.2	0; 0-0	76.5; 212	0; 0-22.5	161; 295.3	70; 17.5-180
**Cleaning Method**	None	399.7; 1191.9	50; 11.3-210	293.8; 804.6	22.5; 11.3-88.8	0; 0	0; 0-0	86.3; 326.6	0; 0-7.5	448.1; 1319.2	40; 0-272.5
	Disinfectant	202.1; 282	95; 30-272.5	78.8; 117.1	30; 15.106.3	0.5; 2.2	0; 0-0	28.3; 69.2	0; 0-22.5	178.1; 348.2	45; 20-140
	Water	269.2; 585.9	60; 20-188.8	70.6; 98.4	25; 11.3-86.3	4.1; 17.9	0; 0-0	87.8; 261.3	0; 0-17.5	138.1; 335.6	45; 10-122.5
	Dry towel	865.6; 2823.8	132.5; 46.3-353.8	513.3; 1846.1	60; 17.5-116.3	8.9; 29.3	0; 0-0	171.1; 484.6	15; 0-40	828.3; 2312.9	235; 47.5-437.5
**Cleaning Frequency**	≤ 1 week	177; 273,2	80; 25-188.8	56.4; 68.2	30; 15-88.8	4.1; 21.2	0; 0-0	27.8; 106.2	0; 0-20	137.8; 255.6	60; 20-132.5
	1- 6 months	297.3; 499.5	117.5; 35-343.8	99.1; 131.8	55; 15-118.8	2.9; 14.7	0; 0-0	85.6; 221.4	0; 0-47.5	242.1; 415.4	75; 20-295
	1 year or never	683.8; 2422.6	40; 10-148.8	462.5; 1581.6	20; 6.3-81.3	1.4; 7.6	0; 0-0	123.6; 446.1	0; 0-7.5	641.1; 2090.1	40; 0-220
**Last Cleaning**	≤10 days	536.1; 2011.2	90; 25-307.5	283.5; 1308.1	30; 15-107.5	4.7; 20.9	0; 0-0	109.7; 360.1	0; 0-20	436.7; 1666.6	60; 10-232.5
	< 1 year	268.5; 524.2	95; 25-270	86.7; 135.5	30; 15-105	3.4; 15.9	0; 0-0	72; 218.8	0; 0-35	235.4; 419.9	50; 20-290
	1 year or never	360; 1125.6	50; 10-170	263.3; 761	22.5; 8.8-66.3	0; 0	0; 0-0	76.7; 308	0; 0-2.5	399.4; 1247.3	35; 0-217.5
**Cover Type**	None	995.9; 2663.3	130; 15-415	561,5; 1730.6	25; 15-120	6.5; 22.1	0; 0-0	199.1; 518.1	0; 0-60	823; 2302.8	70; 0-220
	Flip cover	352.9; 383	265; 38.8-496.3	187.1; 274.3	72.5; 17.5-243.8	0; 0	0; 0-0	31.7; 76.6	5; 0-25	345; 392.5	220; 62.5-525
	Case	165.8; 311.7	70; 26.3-153.8	60.4; 75.4	30; 15-85	2.2; 14.4	0; 0-0	48.6; 157.7	0; 0-20	151.5; 290.6	45; 10-197.5
**Means of Transportation**	Public	289.1; 802	60; 25-205	124.1; 481.2	30; 15-90	1.2; 3.9	0; 0-0	94; 264	0; 0-40	308.8; 874.6	40; 20-230
	Private	488.5; 1751.3	105; 22.5-275	262.9; 1128	30; 12.5-115	5.3; 22.6	0; 0-0	88.4; 327.1	0; 0-20	389; 1436.3	70; 10-275
	Both	47.5; 41.7	42.5; 11.3-88.8	41.3; 49.9	22.5; 8.8-92.5	0; 0	0; 0-0	0; 0	0; 0-0	97.5; 112.7	95; 0-197.5

***Legend**: **CFU**: colony forming units; **HPC**: Heterotrophic Plate Counts; **IQR**: Interquartile Range; **SD**: Standard Deviation.*

Table S2. Multiple linear regression models.

HPC 37 °C									
					Change statistics				
**Model**	**R**	**R square**	**Adjusted R-square**	**Standard Error of the estimate**	**R-square Change**	**F Change**	**df1**	**df2**	**Sig. F Change**
**1**	.261[a]	.068	.050	1257.891	.068	3.796	2	104	**.026***
**2**	.311[b]	.097	.070	1244.460	.029	3.257	1	103	.074
**3**	.329[c]	.108	.064	1248.449	.012	.671	2	101	.513
**4**	.413[d]	.171	.103	1222.504	.062	2.444	3	98	.069

a. Predictors: (constant), Cover type; b. Predictors: (constant), Cover type, Gender; c. Predictors: (constant), Cover type, Gender, Cleaning frequency; d. Predictors: (constant), Cover type, Gender, Cleaning frequency, Cleaning method.

HPC 22 °C									
					Change statistics				
**Model**	**R**	**R square**	**Adjusted R-square**	**Standard Error of the estimate**	**R-square Change**	**F Change**	**df1**	**df2**	**Sig. F Change**
1	.240[a]	.058	.049	797.47375	.058	6.480	1	106	.012*
2	.241[b]	.058	.040	801.13536	.000	.033	1	105	.856

a. Predictors: (constant), Gender; b. Predictors: (constant), Gender, Study Cycle.

Enterococci									
					Change statistics				
**Model**	**R**	**R square**	**Adjusted R-square**	**Standard Error of the estimate**	**R-square Change**	**F Change**	**df1**	**df2**	**Sig. F Change**
1	.228[a]	.052	.034	272.7462	.052	2.859	2	104	.062
2	.304[b]	.093	.048	270.7944	.040	1.502	3	101	.219
3	.327[c]	.107	.053	269.9633	.014	1.623	1	100	.206

a. Predictors: (constant), Cover type; b. Predictors: (constant), Cover type, Cleaning method; c. Predictors: (constant), Cover type, Cleaning method, Gender

Staphylococci									
					Change statistics				
**Model**	**R**	**R square**	**Adjusted R-square**	**Standard Error of the estimate**	**R-square Change**	**F Change**	**df1**	**df2**	**Sig. F Change**
1	.244[a]	.060	.041	1093.4830	.060	3.291	2	104	.041*
2	.355[b]	.126	.083	1069.6518	.067	2.562	3	101	.059
3	.397[c]	.158	.107	1055.4076	.032	3.745	1	100	.056

a. Predictors: (constant), Cover type; b. Predictors: (constant), Cover type, Cleaning method; c. Predictors: (constant), Cover type, Cleaning method, Gender

* Significant values
